# Drug overdose in the ED: a record linkage study examining emergency department ICD-10 coding practices in a cohort of people who inject drugs

**DOI:** 10.1186/s12913-018-3756-8

**Published:** 2018-12-05

**Authors:** Rehana Di Rico, Dhanya Nambiar, Mark Stoové, Paul Dietze

**Affiliations:** 1 Centre For Population Health, Burnet Institute, 85 Commercial Road, Melbourne, VIC 3004 Australia; 2 Department of Epidemiology & Preventive Medicine, Monash University, 553 St Kilda Road, Melbourne, 3004 VIC Australia

**Keywords:** Australia, ICD-10, Poisoning, Overdose, People who inject drugs, Cohort, Record linkage, Emergency services, Surveillance

## Abstract

**Background:**

Drug overdose is a leading cause of mortality and morbidity amongst people who inject drugs (PWID). Drug overdose surveillance typically relies on the International Classification of Diseases (ICD-10) coding system, however its real world utilisation and the implications for surveillance have not been well characterised. This study examines the patterns of ICD-10 coding pertaining to drug overdoses within emergency departments for a cohort of known PWID.

**Methods:**

Cohort data from 688 PWID was linked to statewide emergency department administrative data between January 2008 and June 2013. ICD-10 diagnostic codes pertaining to poisonings by drugs, medicaments and biological substances (T-codes T36-T50) as well as mental and behavioural disorders due to psychoactive substance use (F-codes F10-F19) were examined.

**Results:**

There were 449 unique ED presentations with T or F code mentions contributed by 168 individuals. Nearly half of the T and F codes used were non-specific and did not identify either a drug class (*n* = 160, 36%) or clinical reaction (*n* = 46, 10%) and 8% represented withdrawal states. T and F codes could therefore be used to reasonably infer an illicit drug overdose in only 42% (*n* = 188) of cases. Majority of presentations with T or F overdose codes recorded only one diagnostic code per encounter (83%) and representing multiple-drug overdose (F19.- = 18%) or unidentified substances (T50.9 = 17%) using a single, broad diagnostic code was common.

**Conclusions:**

Reliance on diagnoses alone when examining ED data will likely significantly underestimate incidence of specific drug overdose due to frequent use of non-specific ICD-10 codes and the use of single diagnostic codes to represent polysubstance overdose. Measures to improve coding specificity should be considered and further work is needed to determine the best way to use ED data in overdose surveillance.

## Introduction

People who inject drugs (PWID) experience disproportionate morbidity and mortality related to their drug use [1, 2]. Opioid overdose, in particular, is a leading cause of mortality amongst PWID [1]. Non-fatal opioid overdose is common amongst PWID [3–6] and associated with an increased risk of subsequent fatal overdose [7, 8]. Monitoring overdose occurrence therefore presents a key surveillance target and a potential opportunity to inform interventions to reduce opioid related deaths.

Internationally, there is growing interest in utilising ‘syndromic surveillance’, like that used for infectious disease outbreaks, for drug related harms [9, 10]. The ongoing, systematic gathering of clinical and non-clinical data could provide timely, actionable information on new or emerging drug trends and help facilitate prompt intervention and responses [9, 10]. PWID frequently attend hospital emergency departments (EDs) [11], including for overdose-related presentations [12], and so ED data offer potential utility for surveillance and a rich source of information on drug-related morbidity.

Challenges arise, however, when using health data to study non-fatal opioid overdose due to lack of consensus regarding a case definition of opioid overdose in the literature and a lack of sensitivity and specificity of hospital diagnostic coding practices in detecting these cases [13–18]. The World Health Organization (WHO) International classification of diseases (ICD) is the most commonly employed diagnostic coding method used in health-related administrative data, currently at its tenth revision (ICD-10). The clinical syndrome of any ‘drug overdose’ is typically represented by two broad ranges of codes within the ICD-10; “T36 to T50 Poisoning by drugs, medicaments and biological substances” (‘T-codes’) and “F10 to F19 Mental and behavioural disorders due to psychoactive substance use” (‘F-codes’). Consensus statements released by the WHO Substance Abuse Department recommend that ICD-10 F-codes specifying ‘acute intoxication’ should be used to classify drug overdoses in people with known substance use disorders, reserving the T-codes for poisonings in non-substance dependent people [13, 14]. In practice, however, both T-codes and F-codes are used variably in the clinical setting [15, 16]. These non-standardised coding practices limit the validity and utility of ICD-10 codes in drug overdose surveillance [17, 18]. A thorough understanding of real world ICD-10 coding practices is essential to optimising surveillance efforts.

In this paper we examine F-codes and T-codes in ED attendances among a cohort of known PWID, whose drug use trends [5, 19], rates of ED attendance and reasons for presentation [11, 20] have been characterised previously. Here, we focus specifically on the extent to which different ICD-10 codes are used in coding overdose attendances and consider the implications of this coding for understanding patterns and trends in the presentations of PWID in the ED.

## Methods

### Study population

Six-hundred and eighty-eight PWID were recruited through a combination of respondent-driven sampling, snowball sampling and peer outreach between 2008 and 2010 as part of The Melbourne Injecting Drug User Cohort Study (MIX) [5]. Participants resided in urban Melbourne, the second largest city in Australia, were aged 18 or over, regularly (at least monthly) injected either heroin or methamphetamine in the six months prior to baseline recruitment and had a valid Medicare number (needed to access the universal healthcare system in Australia and used in MIX for record linkage). Most (*n* = 563, 82%) participants reported heroin injection, in isolation or with other substances, within the month prior to recruitment. Further details of the MIX study and baseline cohort characteristics are described elsewhere [5].

### Administrative emergency department data

Australian ED data are collected through separate systems in each state and territory. We accessed clinical data from the Victorian Emergency Minimum Dataset (VEMD), a statewide health database containing de-identified demographic, administrative and clinical details from all 24-h EDs in Victoria. Thirty-eight EDs reported to VEMD during the study period, with 19 EDs located within the greater Melbourne area where most of the study cohort resided. Full descriptions of this database are available elsewhere [21]. Computer software at each reporting ED passively extracts demographic, administrative and clinical data from working clinical documents used in patient care. The software allocates the relevant ICD-10 codes for each encounter by mapping the clinical diagnoses entered by clinicians in their discharge documentation to a significantly abridged set of ICD-10 codes accepted for VEMD reporting (there are approximately 1100 codes in VEMD versus over 60,000 in the full ICD-10 manual). To this end, ED clinicians are not involved in, or directly responsible for, VEMD data collection or ICD-10 code selection [22]. Data are reported to VEMD with only minor input from local administrative staff. This is in contrast to hospital admitted episode data, which are collected by dedicated coding staff at each hospital who are trained to retrospectively review all clinical notes and encode data according to strict criteria, including ICD-10 code selection, specifically for submission to the Victorian Admitted Episode Dataset.

### Record linkage

Cohort data were submitted to the Centre for Victorian Data Linkage (CVDL) for record linkage to VEMD, to identify all ED presentations for the cohort between January 2008 to June 2013. Deterministic record linkage was used, based on 100% match across Medicare number, first three letters of the first name (recorded in VEMD under the variable ‘Medicare Suffix’), date of birth and sex. Linked VEMD data were provided to the researchers, de-identified and encrypted by CVDL, with each participant assigned a unique identifier.

### ICD-10 coding and case definitions

The VEMD records diagnoses according to the International Classification of Diseases, Tenth Revision, modified for use in Australia (ICD-10-AM). The sixth edition was in use during the study period. The VEMD records a primary diagnosis and up to two additional diagnoses (herein referred to as ‘secondary’ and ‘tertiary’ diagnoses). Potential overdoses or poisonings were identified using an ‘any mention’ method [23], which included any mention of the relevant F or T code at any diagnostic level (primary, secondary or tertiary). The F-codes included in this study were F10.0 to F19.9, encompassing “mental and behavioural disorders due to psychoactive substance use”. T-codes included were T36.0 to T50.9, representing “poisoning by drugs, medicaments and biological substances”. T-codes were limited to non-medicinal substances whose primary use was for the feeling they cause [23]. Diagnoses in the T code range T51.0-T65.9 (*n* = 16) represented toxic effects from licit substances such as alcohol, tobacco, as well as other chemicals which could be used for the feeling they cause but are chiefly used for other purposes (eg. organic solvents) and were not included as ‘drug poisons’, in keeping with major coding practices internationally [23]. External cause codes (eg. X42, X62, Y12) characterise overdoses as accidental, intentional or of undetermined intent but are not collected by VEMD and were therefore not included in our analysis. Within the range of included T and F codes, there were subsets pertaining to opioids specifically. Opioid specific T-codes included T40.0-T40.4 and T40.6 and opioid specific F-codes included F11.0-F11.9. The F-code range F19.0 to F19.9 was also explored in regards to polysubstance abuse and co-ingestion.

### Analysis

Analysis was conducted using Stata-14, which includes ICD-10 mapping functionality. Frequencies and proportions were used to describe the range of T and F codes observed in the cohort, based on the highest ranked diagnosis across primary, secondary and tertiary diagnoses. Frequencies and proportions were also used to describe the distribution of relevant ICD-10 codes across the diagnostic levels (primary, secondary or tertiary), as well as any co-mentions of T and F codes within the same encounter.

## Results

There was a total of 3459 ED presentations for the cohort during the five and half year study period. Diagnostic data were missing in 436 records (13%) due to patients leaving after clinical advice about treatment options (without being seen by a definitive service provider) (*n* = 31) or leaving at risk without treatment (*n* = 405) and therefore did not require a diagnostic code to be recorded in VEMD. Ninety-three percent of the remaining 3023 records had only a primary diagnosis recorded, with 7% having a secondary diagnosis and less than 1% having a tertiary diagnosis.

There were 232 T-code mentions across all diagnostic fields in the study period and, when accounting for records with multiple T-codes within the same encounter (*n* = 4), this represented 228 unique presentations (7%) for ‘poisonings’. There were 232 F-code mentions across all diagnostic fields in the study period and, after accounting for cases with multiple F-codes in the same encounter (*n* = 5), this represented 227 (7%) unique presentations for ‘mental and behavioural disorders due to psychoactive substances’. Only six cases had both F and T codes recorded within the same encounter, resulting in a total of 449 unique presentations containing F or T codes, contributed by 168 individuals. One third of all F and T code encounters (*n* = 138, 31%) were due to five individuals with repeat presentations. Demographic details for the 168 individuals at their first F or T code diagnosis in the study period are presented in Table 1.

**Table 1:** Demographic characteristics of 168 participants at their first F or T code diagnosis in ED during the study period

		*N* = 168	%
Mean Age	28.1 years (SD 4.3)	–	–
Proportion by age group (years)	15–19	6	*4*
	20–24	40	*24*
	25–29	66	*39*
	30–34	52	*31*
	35–39	3	*2*
	40–44	1	*1*
Sex	Male	104	*62*
	Female	64	*38*
Preferred Language	English	167	*99*
	Vietnamese	1	*1*
Country of birth	Australia	137	*82*
	Vietnam	8	*5*
	England	3	*2*
	New Zealand	3	*2*
	Other	14	*8*
	Missing	3	*2*
Marital status	Never married	70	*42*
	De Facto	5	*3*
	Married	2	*1*
	Not stated/missing	91	*54*
Usual Accommodation	Private residence (with others)	115	*68*
	Private residence (alone)	14	*8*
	Supported Residential Service (community based)	4	*2*
	Boarding house/Hostel	3	*2*
	Homeless	3	*2*
	Homeless shelter / Other refuge	2	*2*
	Other	2	*2*
	Not stated/missing	25	*15*

Tables 2 and 3 list T and F codes separately, in order of frequency. There were 16 different T-codes and 18 different F-codes representing seven different broad drug classes. Table 2 shows that, among the 228 encounters with poison codes in this PWID cohort, the most frequently recorded T-code was T50.9, a non-specific drug/medication category (*n* = 77, 34%). Table 3 shows there were 139 (61%) ‘acute intoxication’ codes recorded across a range of drug classes, with nearly half of these pertaining to acute alcohol intoxication (*n* = 62, 45%). ‘F19.-: mental and behavioural disorders due to multiple drugs and psychoactive substances’ was the most common diagnostic category amongst the F-codes (*n* = 83, 37%) and included diagnoses pertaining to withdrawal. Examining Tables 2 and 3 together, which represented 449 unique presentations (six presentations involved both T and F codes), T-codes for ‘poisonings’ were more frequent overall compared to F-codes for ‘acute intoxication’ in this cohort of known PWID (51% versus 31% across all F or T code encounters). F-codes were more commonly used to reflect adverse effects of alcohol or polysubstance use, while T-codes were generally used to reflect poisonings due to specific illicit drug classes. Over one third of the F and T codes assigned did not identify a specific drug class (*n* = 160, 36%) and 10% (*n* = 46) had an unspecified clinical presentation which could not distinguish between presentations related to overdose or withdrawal. There were 37 (8%) F-codes which specified withdrawal from substances as opposed to overdose. Therefore, the highest ranked F or T code per encounter could be used to reasonably infer an illicit drug overdose (ie. specifying both an illicit drug class (excludes alcohol) AND a clinical reaction of ‘poisoning’ or ‘acute intoxication’) less than half the time (*n* = 188, 42%).

**Table 2:** Distribution of the 228 T-codes assigned during study period, in order of frequency (highest ranked only^a^)

T36-T50, Poisoning due to:		No. of cases	% of T codes	% of F&T codes
		*n*	*N* = 228	*N* = 449
T50.9	Other and unspecified drugs, medicaments and biological substances^b^	77	33.8	17.1
T40.1	Heroin	37	16.2	8.2
T42.4	Benzodiazepines	33	14.5	7.3
T40.6	Other and unspecified narcotics	16	7.0	3.6
T39.1	4-Aminophenol derivatives	11	4.8	2.4
T43.9	Psychotropic drug, unspecified	11	4.8	2.4
T42.7	Antiepileptic and sedative-hypnotic drugs, unspecified	10	4.4	2.2
T40.2	Other opioids	9	4.0	2.0
T40.3	Methadone	6	2.6	1.3
T43.6	Psychostimulants with abuse potential	4	1.8	0.9
T40.0	Opium	3	1.3	0.7
T40.7	Cannabis (derivatives)	3	1.3	0.7
T41.2	Other and unspecified general anaesthetics	3	1.3	0.7
T40.4	Other synthetic narcotics	2	0.9	0.4
T43.0	Tricyclic and tetracyclic antidepressants	2	0.9	0.4
T40.9	Other and unspecified psychodysleptics [hallucinogens]	1	0.4	0.2

^a^In cases with multiple T codes per encounter (*n* = 4), the highest ranked diagnosis was taken

^b^Diagnosis does not specify drug class

**Table 3:** Distribution of the 227 F-codes assigned during study period, in order of frequency (highest ranked only^a^)

		No. of cases	% of F codes	% of F&T codes
F10.0-F19.9, Mental and behavioural disorders due to use of:		n	*N* = 227	N = 449
F10.0	Alcohol: Acute intoxication	62	27.3	13.8
F19.0	Multiple drugs and use of other psychoactive substances: Acute intoxication^b^	40	17.6	8.9
F19.3	Multiple drugs and use of other psychoactive substances: Withdrawal state^bd^	32	14.1	7.1
F11.0	Opioids: Acute intoxication	21	9.3	4.7
F10.9	Alcohol: Unspecified mental and behavioural disorder^c^	16	7.1	3.6
F19.9	Multiple drugs and use of other psychoactive substances: Unspecified mental and behavioural disorder^bc^	11	4.9	2.4
F13.0	Sedatives or hypnotics: Acute intoxication	10	4.4	2.2
F11.9	Opioids: Unspecified mental and behavioural disorder^c^	9	4.0	2.0
F12.9	Cannabinoids: Unspecified mental and behavioural disorder^c^	5	2.2	1.1
F15.1	Other stimulants, including caffeine: Harmful use	5	2.2	1.1
F10.3	Alcohol: Withdrawal state^d^	4	1.8	0.9
F15.0	Other stimulants, including caffeine: Acute intoxication	4	1.8	0.9
F13.9	Sedatives or hypnotics: Unspecified mental and behavioural disorder^c^	3	1.3	0.7
F10.4	Alcohol: Withdrawal state with delirium^d^	1	0.4	0.2
F12.0	Cannabinoids: Acute intoxication	1	0.4	0.2
F16.9	Hallucinogens: Unspecified mental and behavioural disorder^c^	1	0.4	0.2
F18.0	Volatile solvents: Acute intoxication	1	0.4	0.2
F18.9	Volatile solvents: Unspecified mental and behavioural disorder^c^	1	0.4	0.2

^a^In cases with multiple F codes per encounter *(n = 5)*, the highest ranked diagnosis was taken

^b^Diagnosis does not specify drug class

^c^Diagnosis does not specify clinical syndrome

^d^Diagnosis indicates a withdrawal state (ie. not overdose)

Table 4 excludes codes representing withdrawal states to look more closely at potential overdose related presentations and depicts the diagnostic level (primary, secondary or tertiary) that overdose related codes occur, along with T or F-code co-mentions within a given encounter. A subset of specific ICD-10 code categories were chosen, representing opioid poisonings, mental and behavioural disturbances related to opioids, the highest occurring T and F categories (T50.- and F19.-), as well as all poisonings and all mental and behavioural disturbances due to psychoactive substances. The table shows that when present, F or T codes were frequently primary diagnoses and there was very little overlap, both within or between categories as most overdose-related F or T code presentations received only a primary diagnosis for the encounter (*n* = 346, 83%). Opioid poisonings were more commonly diagnosed than mental and behavioural disturbances due to opioids. Opioid poisoning represented one third of all T-code poisonings (*n* = 74, 32%) and mental and behavioural disorders due to opioids represented 13% (*n* = 30) of F-code diagnoses.

**Table 4:** Number of ED presentations with diagnoses in selected ICD-10 categories, and hierarchical position of the diagnosis within each presentation

		Presentations with relevant code mention (*N* = 3459)		Occurrences as Primary Diagnosis	Occurrences as Secondary Diagnosis	Occurrences as Tertiary Diagnosis	Other T code (T36.- to T50.-) within same encounter	Other F code (F10.- to F19.-) within same encounter
ICD-10 Codes	Description	*n*	%	*n*	*n*	*n*	*n*	*n*
T40.0-T40.4 & T40.6	Opioid poisonings	74	2	72	2	0	2	0
F11.0-F11.9^a^	Mental and behavioural disorders due to opioids	30	1	30	0	0	0	1
T50.9	Poisoning: Other and unspecified drugs, medicaments and biological substances	79	2	73	6	0	2	3
F19.0-F19.9^b^	Mental and behavioural disorders due to multiple drugs and psychoactive substances	52	2	47	4	1	1	1
T36.0-T50.9	ALL: Poisoning by drugs, medicaments and biological substances	228	7	220	12^d^	0	–	6
F10.0-F19.9^c^	ALL: Mental and behavioural disorders due to psychoactive substances	190	6	174	19^e^	1	6	–

^a^Excludes withdrawal states F11.3, F11.4 *(n = 0)*

^b^Excludes withdrawal states F19.3, F19.4 *(n = 33)*

^c^Excludes all withdrawal states (F subdivisions .3 and .4) *(n = 37)*

^d^Overlapping primary diagnosis in same category *(n = 4)*, hence sum of mentions > total number of encounters

^e^Overlapping primary diagnosis in same category *(n = 4),* hence sum of mentions > total number of encounters

## Discussion

We examined how a selection of F and T codes were assigned within ED data for a cohort of 688 known PWID between January 2008 and June 2013. Our findings suggest that ED diagnostic codes used in isolation are not suitable for surveillance of specific drug overdoses in Victoria. There were 449 unique presentations with F and T codes mentioned during the study period. Nearly half of the F and T codes utilised in the cohort were non-specific and either did not identify the drug class involved (36%) or the exact nature of the clinical effect of the drug (overdose versus withdrawal, 10%). As such, only 188 (42%) of F or T codes could be used to reasonably infer an illicit drug overdose event, with the ICD-10 code referencing both an illicit drug class and a clinical reaction of ‘poisoning’ or ‘acute intoxication’. Despite the WHO recommendation to utilise F-codes of ‘acute intoxication’ to characterise overdose in substance dependent persons [14], T-codes for poisonings were more commonly ascribed in this cohort of known PWID, most of whom would be considered clinically substance dependent. A large proportion of ‘acute intoxication’ codes related to alcohol toxicity (45%) rather than illicit substances.

There was a wide dispersion of F and T codes within the cohort, implicating up to seven different drug classes and 18 different substances as primary causes of overdose. Within our cohort, whose patterns of drug use have been well characterised and predominantly involve the use of heroin, other opioids and stimulants [5], this suggests a high degree of misclassification of overdoses and poisonings within the ED. It is well recognised that ED clinicians face numerous diagnostic challenges in classifying drug overdoses. A prospective observational study in a large, tertiary ED in Melbourne, Australia showed that clinicians’ impressions of the substances involved in suspected recreational drug overdose matched laboratory blood tests in 78% of cases, however this figure dropped to 46% when exploring opioids specifically [24]. In a culture where co-ingestion is increasingly common, and pre-hospital naloxone administration (an opioid antagonist) is widely available, the classic opioid poisoning toxidrome of pupillary meiosis, respiratory depression and stupor may not manifest [25]. Toxicological assays are not yet rapid or reliable enough to be clinically useful in acute overdose [26] and precise identification of the causative agent may be clinically irrelevant in the ED context with critically unwell, obtunded or agitated patients who require immediate, symptom directed, supportive care regardless of aetiology [25]. Many overdoses are managed by ambulance services in the community itself without requiring subsequent hospitalisation [27–29], however Victorian ambulance clinical practice guidelines recommend that patients who have not responded within ten minutes to naloxone treatment in the community or have other complicating features should be brought to hospital [30]. This increases the complexity of the ED casemix, further challenging clinicians’ abilities to accurately identify the substances involved in overdoses or poisonings. An additional effect of this on ICD-10 coding may be that, even if an opioid overdose is the underpinning primary event, the clinical diagnosis chosen in ED may pertain to the complicating features necessitating transportation to ED, rather than the overdose itself. For example, a clinician may enter a diagnosis reflecting ‘polysubstance overdose’ because the patient failed to respond to community administered naloxone or record only the medical complications of overdose such as aspiration pneumonia. These coding practices limit the sensitivity and specificity of ICD-10 codes in detecting overdose presentations.

The most common single overdose-related code used was “T50.9: Other and unspecified drugs, medicaments and biological substances”. The official WHO definition states the ‘T50.9’ code is applicable when substances are ‘not elsewhere classified’, listing examples of “acidifying agents, alkalizing agents, immunoglobulin, immunologicals, lipotropic drugs and parathyroid hormones and derivatives” [31]. These substances are highly unlikely to be the most common primary cause of drug overdose in our cohort of PWID with a known predilection for the use of heroin, other opioids and stimulants. Our findings therefore suggest that, in clinical practice, it appears ‘T50.9’ is being adopted as a means to classify encounters where ‘unknown’ or ‘unspecified’ substance(s) are involved. The ‘F19.-’ category, encompassing codes F19.0-F19.9, was the most common diagnostic category seen in the cohort, representing “mental and behavioural disorders due to multiple drugs and psychoactive substances”. Once again, this category is non-specific, referring to ‘multiple drugs’ but not implicating any specific agent or drug class. WHO recommends ‘F19.-’ codes are used in cases when “two or more psychoactive substances are known to be involved but it is impossible to assess which substance contributed most” or “when the exact identity of some or all the psychoactive substances being used is uncertain or unknown” [31]. It is plausible that, despite clinicians’ intensive efforts in ED, the specific substances involved in a drug overdose cannot be identified. However, the high frequency use of this code within a cohort of known PWID with relatively stable drug usage patterns [19] suggests that other contextual factors, such as a lack of understanding of the importance of detailed clinical documentation for surveillance efforts, insensitive data collection tools and competing priorities in a busy ED, may be influencing non-specific coding practices as well. Studies have shown insufficient awareness about ICD-10 coding practices amongst both clinicians and trained coders [16, 22] and there has been criticism that the significantly abridged ICD-10 codes do not adequately capture all substances, particularly new and emerging drugs, forcing clinicians’ diagnoses to be mapped back to inappropriate codes [15]. It is unclear, however, whether increasing the number and specificity of codes available will yield more accurate data. Our study revealed that majority (83%) of overdose encounters with F or T code diagnoses had only a primary diagnosis recorded, despite the option for clinicians to record up to three. In the context of general medical or surgical presentations, a single diagnosis may be appropriate and reflect single organ system dysfunction, however in the context of drug poisoning or overdose, co-ingestion is increasingly common and warrants numerous diagnostic entries to reflect each involved substance. In the busy ED setting, broad non-specific headings such as ‘other and unspecified drugs’ or ‘multiple drugs and psychoactive substances’ may appear convenient and all-encompassing default options to indicate polysubstance overdose, rather than individually entering multiple different agents.

While broad coding practices may be efficient and sufficient for clinical practice, they significantly hamper individual drug overdose surveillance efforts, as non-specific codes are frequently excluded when establishing case definitions. Given then frequent non-specific coding seen within our cohort of primarily opioid users, it is likely that the use of ED data collated on the basis of ICD diagnosis alone would considerably underestimate the incidence of opioid overdose. One way to improve surveillance may be to integrate information from free-text coding available in the VEMD, but this information is not always available (including for this study) and is of unknown reliability or validity [17, 18]. Nevertheless, the question remains as to whether to include non-specific codes within surveillance case definitions, and studies have shown that their inclusion increases sensitivity but reduces specificity of case detection [17, 32]. Potential measures to reduce the utilisation of non-specific codes within the ED could include educating clinicians in data collection processes and implications for surveillance, collaborative review regarding the ICD-10 tool and its appropriateness for drug overdose diagnosis in ED and consideration of the involvement of trained, dedicated coders within EDs (similar to clinical coding used for inpatient records [33]) to relieve the data collection burden from busy clinical staff and potentially improve data quality and consistency.

## Limitations

Limitations of this study include the known shortcomings of administrative data quality in terms of completeness and accuracy, as well as the potential for missed VEMD patient records in the deterministic linking process. The CVDL reviewed their linkage algorithm numerous times to minimise the false negative rate and linked data was closely interrogated by multiple authors to ensure there were no duplicate records. The range of F and T codes included in this study were in line with those commonly used for overdose research and surveillance internationally, however a key limitation for research of this type, using administrative-level data, is that the rate of under- or over- ascertainment of cases cannot be known. Whilst we assume that true overdoses are of sufficient clinical acuity and severity to feature prominently within diagnostic coding for that encounter, it is acknowledged that some cases may have been allocated alternative ICD-10 codes in the ED and therefore not identified by this study. Similarly, it cannot be accurately determined if the encounters with the included T and F codes truly reflected illicit substance overdose. Given the large number of EDs involved, it was not feasible to extract patient level data on toxicology results and vital clinical observations to confirm a clinical overdose for every case. Nonetheless, by utilising a cohort of people who are known to inject drugs (and are therefore at higher risk of illicit drug overdose than the general population) and examining data from multiple EDs statewide, we were able to capture a high number of potential overdose events and describe patterns and trends in the real-life application of overdose-related ICD-10 codes. There is a growing body of research examining the sensitivity and specificity of ICD-10 coding in identifying illicit drug overdose [17, 18] and this will be an area of key relevance for informing surveillance case definitions in future. The limitations of this study are akin to the challenges faced by overdose surveillance efforts at large, and only highlight the importance of further research in this area, to identify opportunities for improving data quality and surveillance targets.

## Conclusion

There is a disconnect between the idealised usage of the ICD-10 codes and its application in everyday practice. Broad, non-specific coding with single primary diagnoses are frequently employed within EDs to classify drug overdoses and therefore reliance on diagnoses alone when examining ED data will likely significantly underestimate incidence of drug overdose for any specific drug. Nonetheless, EDs are at the coal face of serious overdoses and can still play a valuable role in drug surveillance. Further work is needed to determine the best way to use ED data in syndromic surveillance.

## Abbreviations

CVDL: Centre for Victorian Data Linkage
ED: Emergency Department
ICD: International Classification of Diseases
ICD-10: International Classification of Diseases, Tenth Revision
ICD-10-AM: International Classification of Diseases, Tenth Revision, Australian Modification
MIX: Melbourne Injecting Drug User Cohort Study
PWID: People Who Inject Drugs
VEMD: Victorian Emergency Minimum Dataset
WHO: World Health Organization
